# Proteomic Profiling Using a Proximity Extension Assay Panel on Serum Samples Stored at −25°C Over Several Decades

**DOI:** 10.1002/pmic.70145

**Published:** 2026-05-20

**Authors:** Steffan Daniel Bos, Sigrún Helga Lund, Renée Turzanski Fortner, Eivind Hovig, Hilde Langseth

**Affiliations:** ^1^ Department of Research Cancer Registry of Norway Norwegian Institute of Public Health Oslo Norway; ^2^ Department of Microbiology Haukeland University Hospital Bergen Norway; ^3^ deCODE genetics/Amgen Inc. Reykjavik Iceland; ^4^ Faculty of Physical Sciences University of Iceland Reykjavik Iceland; ^5^ Division of Cancer Epidemiology German Cancer Research Center Heidelberg Germany; ^6^ Department of Tumor Biology Institute for Cancer Research Oslo University Hospital Oslo Norway; ^7^ Department of Epidemiology and Biostatistics School of Public Health Imperial College London London UK

**Keywords:** biomarkers, proteomics, serum, stability, storage

## Abstract

The advent of proteomics assays that provide accurate levels of thousands of proteins from relatively small sample volumes opens possibilities for biomarker research in biobanks with valuable samples. In this study, we leverage the applicability of the Olink proximity extension assay to profile serum samples of the Janus Serum Bank, which have been stored at –25°C for several decades. We perform association analysis for the body mass index, sex, smoking status and storage time, and compare these associations against protein associations of these traits in the UK Biobank. We identify significant and plausible associations of proteins levels for the analysed phenotypes. Furthermore, these associations showed a high degree of correlation to the analyses performed on the large‐scale studies by the UK Biobank. The samples obtained in the UK Biobank were collected according to modern standards, with short processing time for the blood samples, and storage at very low temperatures, whereas samples in the Janus Serum Bank were collected over larger timespans, with storage in sub‐optimal temperature and over prolonged periods of time as compared to todays’ standards. This research shows that samples stored under sub‐optimal conditions and over extreme periods of time (up to 50 years) yield reliable protein associations for common traits using a proximity extension assay. Therefore, archived biosamples that were not stored in optimal conditions, including samples of the Janus Serum Bank, remain a highly valuable resource in disease and risk biomarker research using modern and emerging proteomics assay methods.

## 1 Introduction

The recent advent of large‐scale quantitative protein assays that combine DNA sequencing with protein detection has resulted in an affordable and powerful method for identification of associations between proteins and disease conditions and other traits [1, 2]. The resulting high‐quality large‐scale proteomic profiles facilitate an agnostic approach to analysis of protein associations to, for example, cancer, diabetes or lifestyle exposures. This was demonstrated in a large‐scale comparison of the proximity extension assay and aptamer‐based detection of proteins using samples from the UK Biobank (UKB) [3], which showed high degrees of overlap for protein‐phenotype associations between the platforms. Furthermore, the UKB plasma samples and the proximity extension assay were successfully combined with genotype analyses to identify protein quantitative trait loci to investigate the role of these loci and proteins in health and disease [4]. The storage conditions for modern biobanks, including the UKB, are optimised towards the preservation of biomolecules and typically apply storage at −80°C or colder, which is considered to effectively prevent degradation of biomolecules such as DNA, RNA and proteins.

The Janus Serum Bank (JSB) contains prospectively collected serum samples from 318,628 donors, collected between 1972 and 2004 in connection with health examination surveys in Norway. Information on smoking, BMI, physical activity, measured cholesterol, glucose and triglycerides is available from the participants [5]. Yearly, the JSB is linked to the Cancer Registry of Norway, resulting in a complete overview of the cancer incidences in the cohort. As of December 2023, almost 124,000 cancer cases have been registered among the donors. At the time of the initial sample collection and storage, temperatures below −25°C were not feasible for large‐scale sample storage. Whilst the standard of storage at −80°C became established in the 1980's and 1990's, the samples in the JSB have remained stored in a temperature‐controlled freezer at −25°C since collection. This sub‐optimal storage temperature may impact on the sample quality for biomolecules in the serum samples. Quality assurance studies performed on samples stored under the same conditions as the JSB have shown that some analytes are sensitive to freeze‐thaw cycles and/or may be affected by increasing time in storage [6, 7]. The long storage time and sub‐optimal temperature may have impacted the usability of the samples in the JSB in large‐scale proteomic studies. Therefore, we aim to investigate if the JSB samples are of sufficient quality to produce high‐quality proteomic profiles using a proximity extension assay and perform a comparison against the data of the large‐scale UKB samples stored in more optimal conditions and with shorter storage time, which were assayed on the same platform.

## 2 Materials and Methods

### 2.1 Sample Collection

The JSB consists of over 700,000 serum samples collected from 318,628 donors between 1972 and 2004. For this study, we selected 348 deceased individuals who were never diagnosed with cancer with a follow up of at least 10 years from serum sampling. The donors selected from the available collection were balanced for sex (50% males, 50% females), smoking status (50% current, 50% never or not currently smoking) and BMI (33% each from the low, medium and high tertiles, where the tertiles are based on the entire JSB [5]). The sample and data collection period spans 32 years and has differences in the blood serum collection procedure used. For the collection between 1972 and 1978 (Group 1), the samples were collected in 10 mL glass blood tubes containing 10 mg of iodoacetate. The iodoacetate causes inhibition of enzymes including glyceraldehyde‐3‐phosphatase, arresting the glycolysis pathway, intended to stabilize the glucose sugar levels. Between 1979 and 1986 (Group 2), the blood samples were collected in 10 mL glass tubes without any additions, and between 1987 and 2004 (Group 3) the blood samples were collected in glass collection tubes containing a gel clot to facilitate easier separation of the serum fraction. During all collection periods, samples were allowed to clot for 60 min before 10 min of 1300 g centrifugation at room temperature. The serum was pipetted over to a glass container with screwcap cork and stored at −25°C. The serum samples are stored in a single temperature‐controlled and alarmed freezer room.

### 2.2 Sample Preparation and Distribution Over Assay Plates

For selected donors, the serum sample vials were drawn from the freezer and thawed. After thorough mixing by repeated aspiration, a 100 µL aliquot was transferred to a matrix tube (Thermo Fisher Scientific, Product Code.10089692, Germany) and shipped on dry‐ice to the laboratory facility of deCODE genetics, Reykjavik, Iceland. Samples were placed randomly over the four 96‐well assay plates with a balanced distribution of BMI tertiles, collection group, sex and the donor smoking status. On each of the four plates, eight random positions were used for the internal assay controls. The distribution of serum samples and control positions is visualised in Figure S1A–D for respectively BMI, collection group, sex and smoking status. Four samples originating from the collection Group 1 were assayed in duplicate, with each duplicate pair being randomly placed on one of the four plates.

### 2.3 Proximity Extension Protein Assay

The proximity extension assay panel ‘Olink Explore’ (Olink—Part of Thermo Fisher Scientific, Uppsala, Sweden) was used according to the manufacturers protocol, resulting in data for 2939 targets (representing 2923 unique proteins). For an extensive description of the procedure and quality assurance used in the laboratory, see the publication by Eldjarn et al. [3]. Duplicated samples were collapsed into one pseudo‐sample by taking the mean of the normalised protein expression (NPX) values for the two measurements.

### 2.4 Assay Data Quality Assurance—Principal Component Analyses (PCA)

PCA were performed on the NPX values for the assay targets with the 30% highest variance. One PCA was performed for all samples, as well as three group‐specific stratified PCAs for Group 1, Group 2 and Group 3. The first two principal components were used to define outliers that deviated more than 5 standard deviations from the mean for a single principal component (Figure S2A for the overall PCA and Figure S2B–D for PCA performed stratified Group 1 through Group 3). One sample was identified as an outlier in the Group 1 specific principal component 2 and this sample was excluded from all subsequent analyses (Figure S2B).

### 2.5 Statistics

To identify assay targets associated to the phenotypes investigated, a linear model was fit in which the protein NPX value was the outcome and the input variables age at blood draw (quantitative in years), BMI (quantitative in kg/m^2^), sampling group (categorical), sex (binary categorical), smoking status (binary categorical) and days in storage (quantitative in years). The effects sizes reported are the beta for quantitative variables BMI and storages time, and the odds ratio between categories for the binary phenotypes sex ('female' as reference category) and smoking status ('not currently smoking' as reference category). For differences between the three collection groups, we only report the *p* values for the associations. A specific analysis was performed to identify the assay targets that were most differentially expressed between Group 1 and the other collections Group 2 and Group 3 combined. To obtain association statistics for the storage time across the different collection Groups, we performed a fixed effects meta‐analysis of the effect size and *p* value on the years in storage association statistics assessed within the stratified collection Groups 1–3. To compare the proteins with the highest abundance against the proteins with the lowest abundance we divided the proteins into quintiles based on the 5%‐trimmed mean NPX values. Where applicable, associations are corrected for multiple testing according to Benjamini and Hochberg with a false discovery rate (fdr) of 0.05.

Correlations between effect sizes for the collection groups were investigated for assay targets that showed a significant association (nominal *p* value < 0.05) in both collection group strata and between the JSB overall and the UKB study. The Spearman correlation coefficients are provided as the rho (*ρ*), nominal *p* values for the respective correlations are provided.

All analyses were performed in R 4.3.2.

## 3 Results

### 3.1 Sample Characteristics

The characteristics of the samples that passed quality control (347 out of 348 samples assayed, see materials and methods) are provided in Table 1.

**TABLE 1:** Sample characteristics for 347 samples in the proteomics analysis of long‐term stored serum. The statistics are provided for all samples in the study and for the three sampling subsets of the total study: Group 1, Group 2 and Group 3.

	All (*N* = 347)	Group 1 (*N* = 115)	Group 2 (*N* = 116)	Group 3 (*N* = 116)
Female, *N* (%)	174 (50%)	58 (50%)	59 (51%)	57 (49%)
Age in years, mean (range)	40.2 (39–41)	40.1 (39–41)	40.3 (39–41)	40.3 (39–41)
BMI, mean (range)	25.6 (15.6–42.3)	25.5 (17.5–40.3)	25.5 (17.6–36.7)	25.6 (15.6–42.3)
Currently smoking, *N* (%)	176 (51%)	59 (51%)	59 (51%)	58 (50%)
Storage years, range	40 (32–51)	48 (45–51)	39 (37–42)	34 (32–36)

### 3.2 Comparison of Collection Groups

In total, 2939 targets were analysed for 347 individual donors after exclusion of one sample with outlier values (methods). The principal component analysis for all samples combined (Figure S2A) shows samples in the collection Group 1 cluster distinct from Group 2 and Group 3, whilst the clusters for the latter two groups overlapped. The five most significant differentially expressed assay targets between Group 1 and the other collections Group 2 and Group 3 considered together are provided in Table 2.

**TABLE 2:** Statistics for the top five most differentially expressed assay targets between Group 1 and Groups 2 and 3 based on the association *p* value. The respective statistics for all assay targets are provided in Table S1.

Rank	Target^*^	Coef^**^	*p* value
1	GNPDA2	3.72	1.6 × 10^−97^
2	QDPR	4.32	9.9 × 10^−81^
3	SHMT1	5.19	9.4 × 10^−72^
4	TPK1	3.38	2.5 × 10^−63^
5	APOA2	2.46	2.5 × 10^−59^

* Target indicates the protein annotated to the Olink assay OID.

** *Coef*: Effect size in NPX‐units between Group 1 and Groups 2 and 3.

### 3.3 Assay Target Associations to BMI, Sex, Smoking Status and Storage Time

Statistics for the phenotype associations to BMI, sex, smoking status and storage time are provided in Table S1; for these analyses, the model included the collection group and donor age. For each phenotype, the five most strongly associated assay targets are listed in Table 3. The largest number of significant protein associations were observed for storage time with 1540 associated targets (fdr < 0.05; 52% of the total of 2939 targets analysed). Sex had 949 associated targets (fdr < 0.05; 32% of the total of 2939 targets analysed). Sex had the strongest protein associations when considering the *p* values. Smoking had 672 associated targets (fdr < 0.05; 23 % of all analysed targets) and BMI had 602 associated assay targets (fdr < 0.05; 20% of all analysed targets). We did not observe differences in the fractions of significant associations to storage time when comparing the proteins in the lowest quintile and highest quintile (Table S2).

**TABLE 3:** Statistics for the top five most associated assay targets based on the *p* value (FDR‐corrected) for the phenotypes BMI, sex, smoking status and storage time. Statistics for phenotype associations for all assay targets are provided in Table S1.

	BMI (kg/m^2^)			Sex (ref. '*Female'*)			Smoking (ref. '*Not smoking'*)			Storage (years)		
Rank	Target^*^	Coef^**^	*p* value	Target^*^	Coef^**^	*p* value	Target^*^	Coef^**^	*p* value	Target^*^	Coef^**^	*p* value
1	LEP	0.23	6.9 × 10^−61^	INSL3	2.13	4.3 × 10^−126^	ALPP	3.1	1.2 × 10^−72^	PRAP1	−0.18	1.2 × 10^−11^
2	GHR	0.08	3.7 × 10^−29^	KLK3	2.71	6.1 × 10^−103^	LAMP3	1.3	1.5 × 10^−65^	ITGA6	0.18	1.7 × 10^−11^
3	FABP4	0.11	1.5 × 10^−28^	SPINT3	6.67	1.2 × 10^−96^	SCGB3A2	1.7	1.7 × 10^−51^	YES1	0.26	2.4 × 10^−11^
4	PLAT	0.09	1.2 × 10^−21^	EDDM3B	1.15	5.1 × 10^−86^	PIGR	0.9	9.2 × 10^−41^	BAG3	−0.22	3.3 × 10^−11^
5	GPD1	0.09	4.9 × 10^−20^	ACRV1	1.95	1.5 × 10^−70^	EDIL3	0.8	1.2 × 10^−34^	CGREF1	−0.13	4.1 × 10^−11^

*Note: p* values provided are FDR corrected.

**Abbreviation**: ref., reference category.

* Target indicates the protein annotated to the Olink assay OID.

** *Coef*: For BMI the coefficient per 1‐point increase in BMI, for the binary categorical variables the mean NPX difference between the levels of the variable and for storage time the coefficient per year increase in storage time.

### 3.4 Stratified Analyses for Groups 1, 2 and 3

**FIGURE 1:**
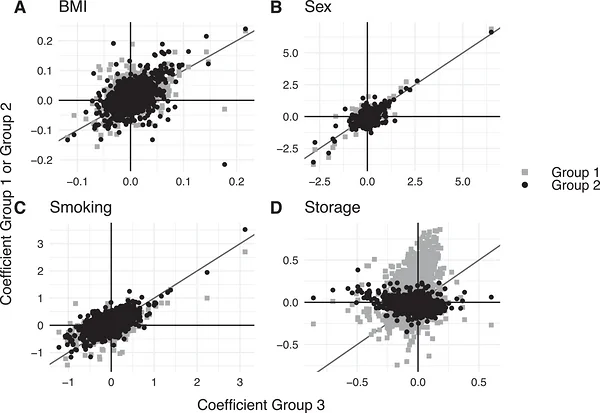
Coefficient plots for (A) BMI, (B) sex, (C) smoking status and (D) storage time. The association coefficients for Group 1 (grey squares) and Group 2 (black circles) on the *y*‐axes are plotted against the association coefficient for Group 3 (the dark‐grey line in the plot depicts a theoretically perfect correlation).

To investigate whether the associations observed in the analyses with samples considered combined are also observed within the three collection groups separately, analyses stratified by collection groups were performed. With exception of storage time, the results from these analyses show a high degree of similarity for the associations’ effect sizes, whereas the level of significance was predominantly more modest. Figure 1A–D shows the effect sizes of the collection Group 1 and Group 2 compared against the collection Group 3 for respectively BMI (Figure 1A); sex (Figure 1B); smoking status (Figure 1C) and storage time (Figure 1D). The Spearman correlation coefficients (Table 4) for the coefficients for significant protein‐phenotype associations (nominal *p* value < 0.05) for BMI, sex and smoking status were strong to very strong, whereas storage time was not strongly correlated across the collection groups and showed an opposite direction of effect between Groups 2 and 3 (Table 4).

**TABLE 4:** Spearman correlation coefficients (rho) of the effect sizes for BMI, sex, smoking status and storage time for analyses stratified by collection Groups. The coefficients are calculated for all association coefficients that are significant (nominal *p* value < 0.05) in both strata.

Phenotype	Group 1 vs. Group 3	Group 2 vs. Group 3
	rho (*p* value, N proteins)	rho (*p* value, N proteins)
BMI	0.86 (< 0.001, 261)	0.85 (< 0.001, 282)
Sex	0.81 (< 0.001, 348)	0.92 (< 0.001, 258)
Smoking status	0.90 (< 0.001, 191)	0.91 (< 0.001, 396)
Storage	0.44 (< 0.001, 426)	−0.53 (< 0.001, 78)

### 3.5 Comparisons Against UKB Data

**FIGURE 2:**
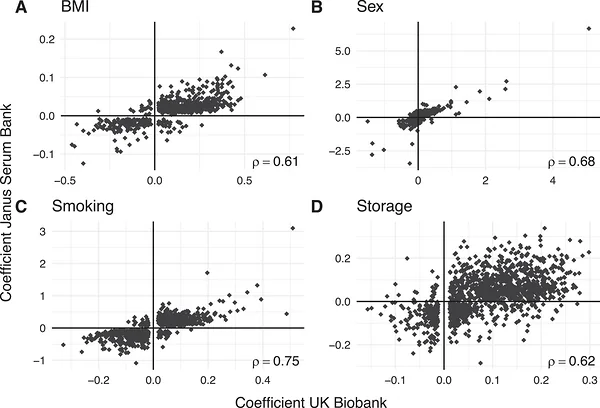
Comparison of the association coefficients of the phenotype associations reported in the large‐scale UK biobank studies (*x*‐axis) and the current study samples (*y*‐axis). Association coefficients are plotted for assay targets with association *p* values below 0.05 in the current study and association *p* values below 0.05 in the UK biobank study, irrespective of the direction of effect. The Spearman rank correlation coefficients (rho) are provided for the protein coefficients between the JSB and UKB. (A) BMI, (B) sex, (C) smoking status and (D) storage time.

For the significant associations to the phenotypes BMI, sex, smoking status and storage time, we compared the association coefficients against the coefficients reported in the UKB [3, 4]. As illustrated in Figure 2A–D and listed in Table 5, we observe good correlations of the coefficients from our study when compared against the coefficients reported by the UKB for respectively BMI, sex, smoking status and storage time. We also compared the associations’ effect size for analyses stratified for Group 1 through Group 3 against the respective coefficients reported in the UKB, provided in Figure S3A–L and Table S3. These show the same trends for BMI, sex and smoking status, whereas storage time is only positively correlated for the samples with the longest storage time, a weak negative correlation for the samples with the intermediate storage time and no correlation for samples with the shortest storage time.

**TABLE 5:** Spearman correlation coefficients (rho) between the effect sizes of respectively BMI, sex, smoking status and storage time for the JSB and the reference data based on samples of the UKB [3, 4]. Correlations were calculated for the coefficients of those assay targets that were nominally significant (*p* value < 0.05) in the JSB analyses and the UKB. The visual representation of the effect sizes of the phenotypes investigated in current study and the UKB are provided in Figure 2.

Phenotype	Current study vs. UKB
	rho (*p* value, N proteins)
BMI	0.61 (< 0.001, 811)
Sex	0.68 (< 0.001, 1110)
Smoking status	0.75 (< 0.001, 829)
Storage	0.62 (< 0.001, 1292)

## 4 Discussion

We present proteomic profiles obtained from 347 samples, balanced for the phenotypes BMI (quantitative with equal representation from the tertiles low/medium/high), sex (female/male) and smoking (smoking/not smoking) and from three sample collection periods in the JSB. At the time of the proteomics assay the samples had been stored at −25°C between 32 and 51 years (mean storage time 40 years). Our study shows a limited impact of storage over several decades and at sub‐optimal freezing temperatures for the phenotypes BMI, sex and smoking status. The effect sizes for protein associations to BMI, sex and smoking status were similar to those observed in the recent collection by the UKB, in which the samples were stored for a shorter period and at substantially lower freezing temperatures. We furthermore observed good correlation between protein associations for the storage time when comparing the data from the JSB to data from the UKB, although this appears to be driven by a relatively strong correlation between the storage time protein associations of the samples in the JSB with the longest storage time (Group 1). The storage time associations between the other two collection groups showed a weak inverse correlation (Group 2) or no correlation at all (Group 3). We cannot rule out that the differences in correlations for storage time are related to collection procedures or to biological or other technical features that differed between the collection periods.

We investigated common, well‐covered phenotypes in the JSB which had similar data available in the UKB [3, 4] to validate findings against a large‐scale, high‐quality reference dataset. Furthermore, the associations observed for BMI, sex and smoking status reflect credible biological processes involved in the analysed phenotypes. The most strongly associated assay target for BMI is annotated to leptin, a known fat‐derived hormone, followed by the assay targeting growth hormone receptor, a protein involvement in stimulating lipolysis. The most strongly associated assay target for sex targets Insulin‐like 3, which is primarily excreted by Leydig cells in the testes. The second strongest associated assay target is annotated to kallikrein‐related peptidase 3 is a protease synthesised by the prostate gland. For smoking, the strongest assay target association was observed for the protein target ALPP, a protein that is reported to be significantly elevated in smokers [8]. The second most associated assay target for smoking status is annotated to LAMP3, a membrane protein that plays a role in the lysosome and which has been shown to associate to smoking status previously [9]. Whilst the most proteins were affected by storage time, these associations had the lowest levels of significance. The directions of effect were both higher (49% of significant associations) and lower (51% of significant associations) and irrespective of the protein abundance, indicating that overall protein abundance does not impact the stability in storage. For comparison, the directions of effect were predominantly higher protein levels for increasing storage time in the UKB (80% of significant associations). We note that between the JSB and UKB there are considerable differences in storage conditions (respectively at −25°C and −80°C) and storage time (respectively 32–51 years and 11–16 years), which may result in differing storage time associations.

Between the collection groups, the most striking difference was observed between collection Group 1 and the other two collection groups. During the collection in Group 1, blood was collected in collection tubes containing the additive iodoacetate. This chemical reacts with thiol groups in enzymes, thereby inactivating enzyme activity. This results in inhibition of enzymes in the glycolysis pathway, thereby preserving the glucose levels in the samples [10]. The protein targets that differed most substantially between Group 1 and other two collections Group 2 and Group 3 most likely reflect the impact of the addition of this chemical compound during the Group 1 collection. Given the relatively short window of time between exposure to iodoacetate and the extraction and freezing of the serum, changes in the levels of these enzymes are unlikely and rather a reflection of the irreversible molecular changes of enzymes induced by the iodoacetate. Iodoacetate results in alkylation of the cysteine residues at the enzymes active sites and as a result of this, antigen recognition by the protein assay may be disturbed. We cannot confirm this hypothesis in the current experiment, and further investigation of this is needed to elucidate the cause of the observed differences in enzyme protein values in this assay. Furthermore, other potential causes of differences within the collection procedures which were not recorded during the collection of the samples such as variations in collection labs, single‐use glass and plastics or other technical changes may underlie the observed differences.

Despite the clear difference for the samples subjected to the iodoacetate treatment in the oldest collection group, we observe similar phenotype associations to BMI, sex and smoking when considering the strongest associations in the analyses stratified for the three collection groups. The more modest levels of significance for the stratified analyses are most likely attributable to the loss of power when analysing smaller sample sets. Overall, the stratified analyses indicates that the protein associations observed are robust with respect to sample pre‐treatment and storage time. The storage time showed varying associations to storage time between the collection groups, without a clear cause for this observation.

We compared the effect sizes from our study to those that were reported in large sample sizes from the UKB [3, 4]. For the collection of the UKB, the collection procedure and storage are optimised to todays’ standards, with highly standardised sample collection procedures, prompt sample processing upon collection and storage at −80°C. The collection and storage conditions as well as the total number of samples in the UKB study, therefore, warrants that this collection can be considered a good reference for the associations of BMI, sex and smoking status. Whilst the proteomics profiles of the UKB are obtained using plasma, our study is based on serum samples. A recent study using an earlier version of the Olink Explore panel shows that the protein contents of plasma and serum are strongly correlated for this assay [11]. The coefficients obtained from our samples show highly significant correlations to the effect coefficients in these large‐scale UKB studies, indicating that suboptimal storage conditions and storage up to 51 years at −25°C did not have a profound negative impact on the usability of these samples for proteomics assays with the proximity extension assay technology. With a smaller sample size in our study and the potential differences in the collection of the phenotypes in the current study and the UKB differ, we were able to validate many of the reported protein‐phenotype associations, particularly for the strongest effect sizes between proteins and phenotypes. Using proteomics data obtained from samples in the UKB linked to cancer data, prospective cancer biomarkers were identified by Papier et al. [12]. Our current study indicates that also older sample biobanks such as the JSB are potentially valuable resources for cancer biomarker research.

We conclude that serum samples stored at −25°C for over several decades and collected using different sampling procedures yield reliable phenotype associations for BMI, sex and smoking status when using a protein proximity extension assay. The findings in the samples of the JSB indicate that biological samples which have experienced prolonged storage and/or storage at suboptimal temperatures can be used with modern proteomic assays for biomarker research.

## Author Contributions

All authors have contributed to conceiving and/or designing the study, data acquirement and interpreting the results, drafted the article and approved the final version. Data quality control and analyses were performed by S.D.B. and S.H.L. All co‐authors have access to the data and analyses scripts and agree to be accountable for all aspects of the work in ensuring that questions related to the accuracy or integrity of any part of the work are appropriately investigated and resolved.

## Funding

This study did not receive financial assistance and has been funded through in‐kind efforts between The Cancer Registry of Norway (Sample handling and data linkage) and deCODE genetics (Olink Explore assay and data QC).

## Ethics Statement

The study was presented to the Southeastern Norway regional ethical committee and deemed to qualify as a quality assurance study for which no ethical approval is required. The communication regarding this is available upon request (in Norwegian).

## Conflicts of Interest

The authors declare no conflicts of interest.

## Supporting information




**Supporting File 1**: pmic70145‐sup‐0001‐SuppMat.pdf.

**Supporting File 2**: pmic70145‐sup‐0002‐TableS1.xlsx.

**Supporting File 3**: pmic70145‐sup‐0003‐FigureS3.pdf.

## Data Availability

Summary statistics for all protein targets are provided in Table S1. The individual‐level protein data and analysis code that support the findings of this study are available on request from the corresponding author with the prerequisite of a signed data transfer agreement between the Norwegian Institute of Public Health and the researchers’ host institutions. The sharing of data requires assessment by the local data protection officer at the Norwegian Institute of Public Health and approval from the Janus Serum Bank steering committee.

## References

[1] L. Wik , N. Nordberg , J. Broberg , et al., “Proximity Extension Assay in Combination With Next‐Generation Sequencing for High‐Throughput Proteome‐Wide Analysis,” Molecular & Cellular Proteomics 20 (2021): 100168, 10.1016/j.mcpro.2021.100168. 34715355 PMC8633680

[2] L. Gold , D. Ayers , J. Bertino , et al., “Aptamer‐Based Multiplexed Proteomic Technology for Biomarker Discovery,” PLoS One 5, no. 12 (2010): 15004, 10.1371/journal.pone.0015004. PMC3000457 21165148

[3] G. H. Eldjarn , E. Ferkingstad , S. H. Lund , et al., “Large‐Scale Plasma Proteomics Comparisons Through Genetics and Disease Associations,” Nature 622, no. 7982 (2023): 348–358, 10.1038/s41586-023-06563-x. 37794188 PMC10567571

[4] B. B. Sun , J. Chiou , M. Traylor , et al., “Plasma Proteomic Associations With Genetics and Health in the UK Biobank,” Nature 622, no. 7982 (2023): 329–338, 10.1038/s41586-023-06592-6. 37794186 PMC10567551

[5] H. Langseth , R. E. Gislefoss , J. I. Martinsen , J. Dillner , and G. Ursin , “Cohort Profile: The Janus Serum Bank Cohort in Norway,” International Journal of Epidemiology 46, no. 2 (2017): 403–404. 27063606 10.1093/ije/dyw027

[6] S. D. Bos , M. Lauritzen , R. E. Gislefoss , N. Stoer , O. I. Klingenberg , and H. Langseth , “Effect of Storage Time up to Nine Years at −25°C on 15 Selected Biochemical Serum Components,” Biopreservation and Biobanking 24 (2026): 136–144, 10.1177/19475535251371418. 40932622

[7] R. E. Gislefoss , M. Lauritzen , H. Langseth , and L. Morkrid , “Effect of Multiple Freeze‐Thaw Cycles on Selected Biochemical Serum Components,” Clinical Chemistry and Laboratory Medicine (CCLM) 55, no. 7 (2017): 967–973, 10.1515/cclm-2016-0892. 27987362

[8] O. S. Nielsen , A. J. Munro , W. Duncan , et al., “Is Placental Alkaline Phosphatase (PLAP) a Useful Marker for Seminoma?,” European Journal of Cancer and Clinical Oncology 26, no. 10 (1990): 1049–1054, 10.1016/0277-5379(90)90049-Y. 2148879

[9] H. W. Grievink , V. Smit , B. W. Huisman , et al., “Cardiovascular Risk Factors: The Effects of Ageing and Smoking on the Immune System, an Observational Clinical Study,” Frontiers in Immunology 13 (2022): 968815, 10.3389/fimmu.2022.968815. 36189218 PMC9519851

[10] M. I. Sabri and S. Ochs , “Inhibition of Glyceraldehyde‐3‐Phosphate Dehydrogenase in Mammalian Nerve by Iodoacetic Acid,” Journal of Neurochemistry 18, no. 8 (1971): 1509–1514, 10.1111/j.1471-4159.1971.tb00013.x. 4398402

[11] R. Shraim , C. Diorio , S. W. Canna , et al., “A Method for Comparing Proteins Measured in Serum and Plasma by Olink Proximity Extension Assay,” Molecular & Cellular Proteomics 24, no. 7 (2025): 101000, 10.1016/j.mcpro.2025.101000. 40441439 PMC12418474

[12] K. Papier , J. R. Atkins , T. Y. N. Tong , et al., “Identifying Proteomic Risk Factors for Cancer Using Prospective and Exome Analyses of 1463 Circulating Proteins and Risk of 19 Cancers in the UK Biobank,” Nature Communications 15, no. 1 (2024): 4010, 10.1038/s41467-024-48017-6. PMC11096312 38750076

