# Supplementary material for: Proteomic Profiling Using a Proximity Extension Assay Panel on Serum Samples Stored at −25°C Over Several Decades
Source: Proteomics. 2026 May 20;26(9):106–12. doi: 10.1002/pmic.70145 (PMC13519378; doi:10.1002/pmic.70145)
Supplement: Supplementary file 1 [file PMIC-26--s002.pdf]

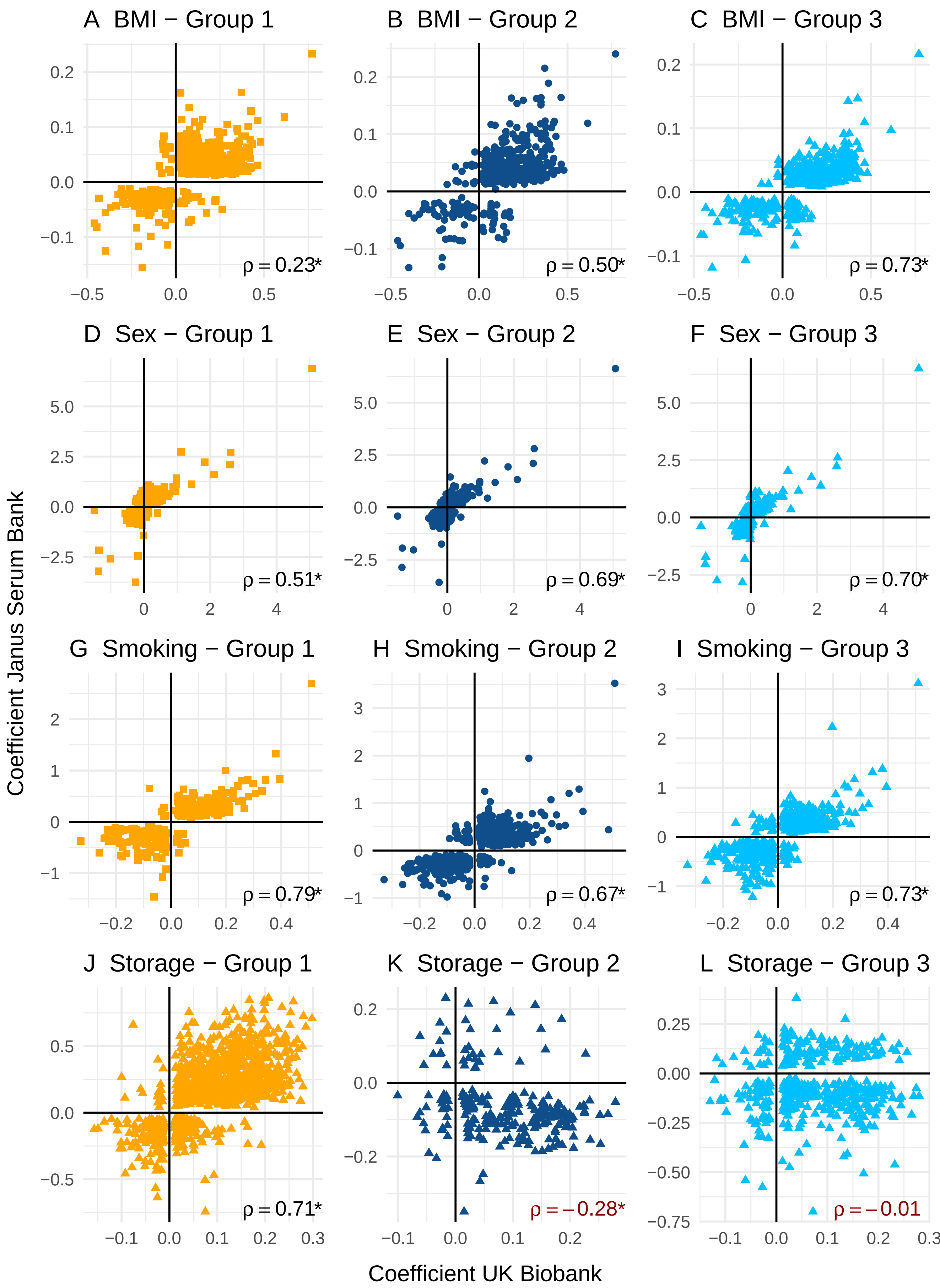

A BMI − Group 1
B BMI − Group 2
C BMI − Group 3
D Sex − Group 1
E Sex − Group 2
F Sex − Group 3
G Smoking − Group 1
H Smoking − Group 2
I Smoking − Group 3
J Storage − Group 1
K Storage − Group 2
L Storage − Group 3
ρ = 0.23*
ρ = 0.50*
ρ = 0.73*
ρ = 0.51*
ρ = 0.69*
ρ = 0.70*
ρ = 0.79*
ρ = 0.67*
ρ = 0.73*
ρ = 0.71*
ρ = −0.28*
ρ = −0.01
Coefficient Janus Serum Bank
Coefficient UK Biobank
