# Supplementary material for: Proteomic Profiling Using a Proximity Extension Assay Panel on Serum Samples Stored at −25°C Over Several Decades
Source: Proteomics. 2026 May 20;26(9):106–12. doi: 10.1002/pmic.70145 (PMC13519378; doi:10.1002/pmic.70145)
Supplement: Supplementary file 2 [file PMIC-26--s003.pdf]

Supplementary Material

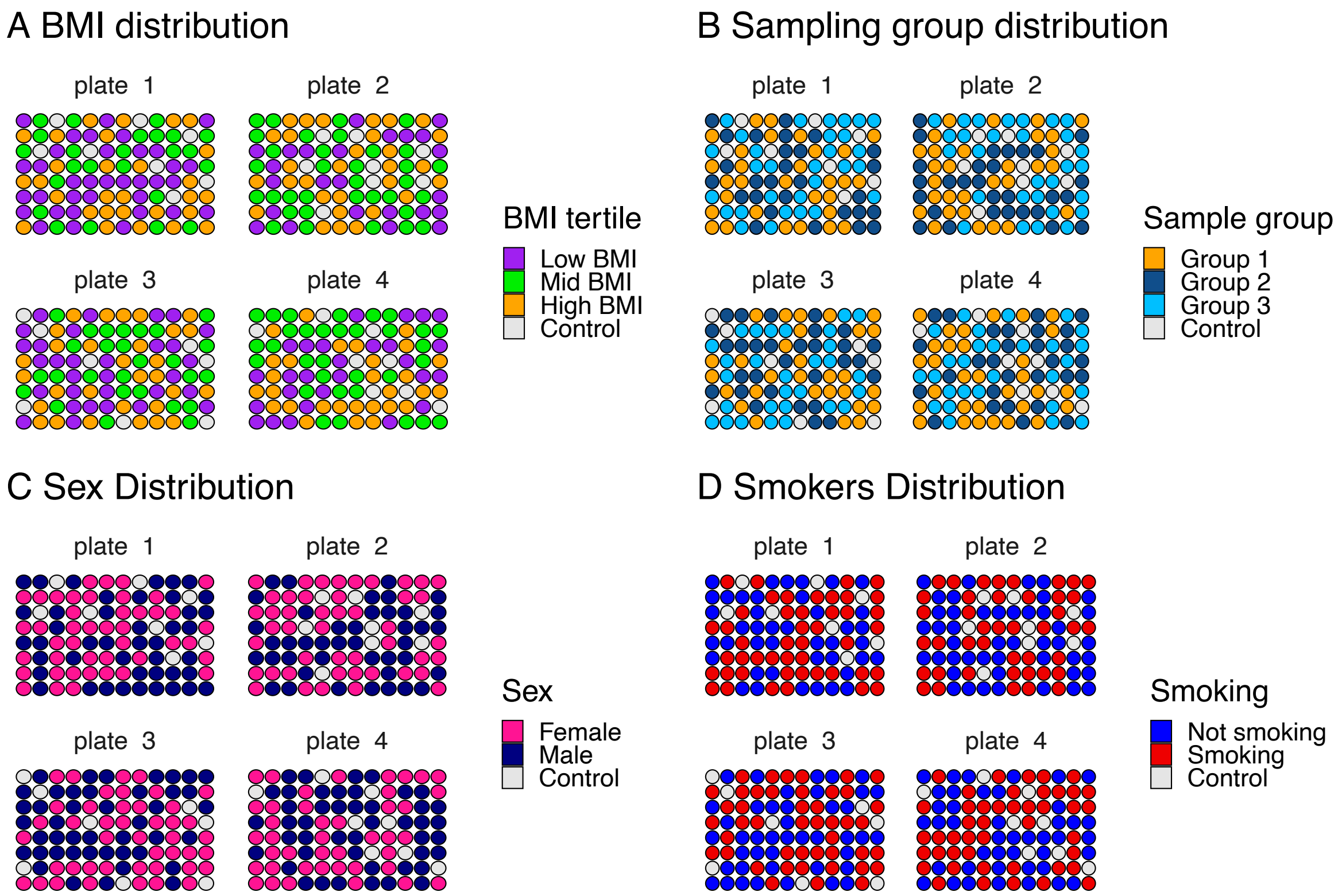

Supplementary Figure 1. Sample distribution over the assay plates. For each plate the grey wells indicate the randomly distributed eight internal assay control positions (gray). A) BMI tertiles, orange - highest BMI tertile; blue - middle BMI tertile; purple - lowest BMI tertile. B) Sampling Groups, orange – Group 1 (1972-1978); dark blue – Group 2 (1979-1986) and light blue – Group 3 (1987-2004). C) Sex, pink – female; blue - male. D) Smoking status, blue - not smoking; red - smoking.

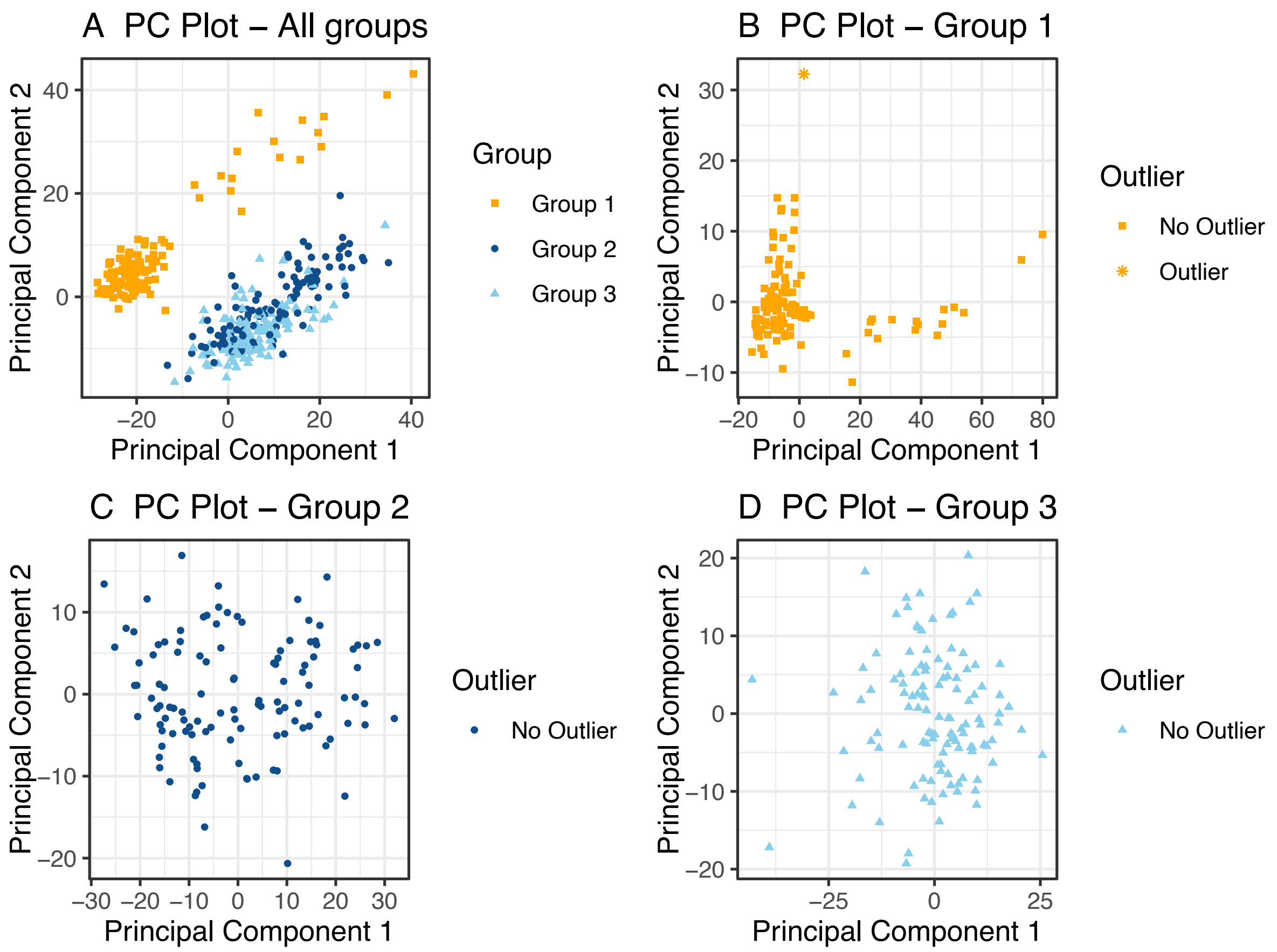

Supplementary Figure 2. Principal component analysis plots of the first two principal components. The x-axis displays the principal component 1 scores for individual samples, the y-axis displays the principal component 2 scores for individual samples. A) Principal component of all 348 samples shows clustering of the Group 1 samples (orange squares) distinct from the Group 2 (dark blue rounds) and Group 3 (light blue triangles) samples. The Group 2 and Group 3 samples show overlapping clusters in the principal component space for component 1 and component 2. Principal component 1 explains 37 % of the total variation, whereas principal component 2 explains 3.8 % of the total variation. B) The Group 1 specific principal component analysis shows one outlier sample (star shape) that is more than 5 standard deviations away from the mean for component 2. C) The Group 2 specific principal component analysis shows no outlier samples. D) The Group 3 specific principal component analysis shows no outlier samples.

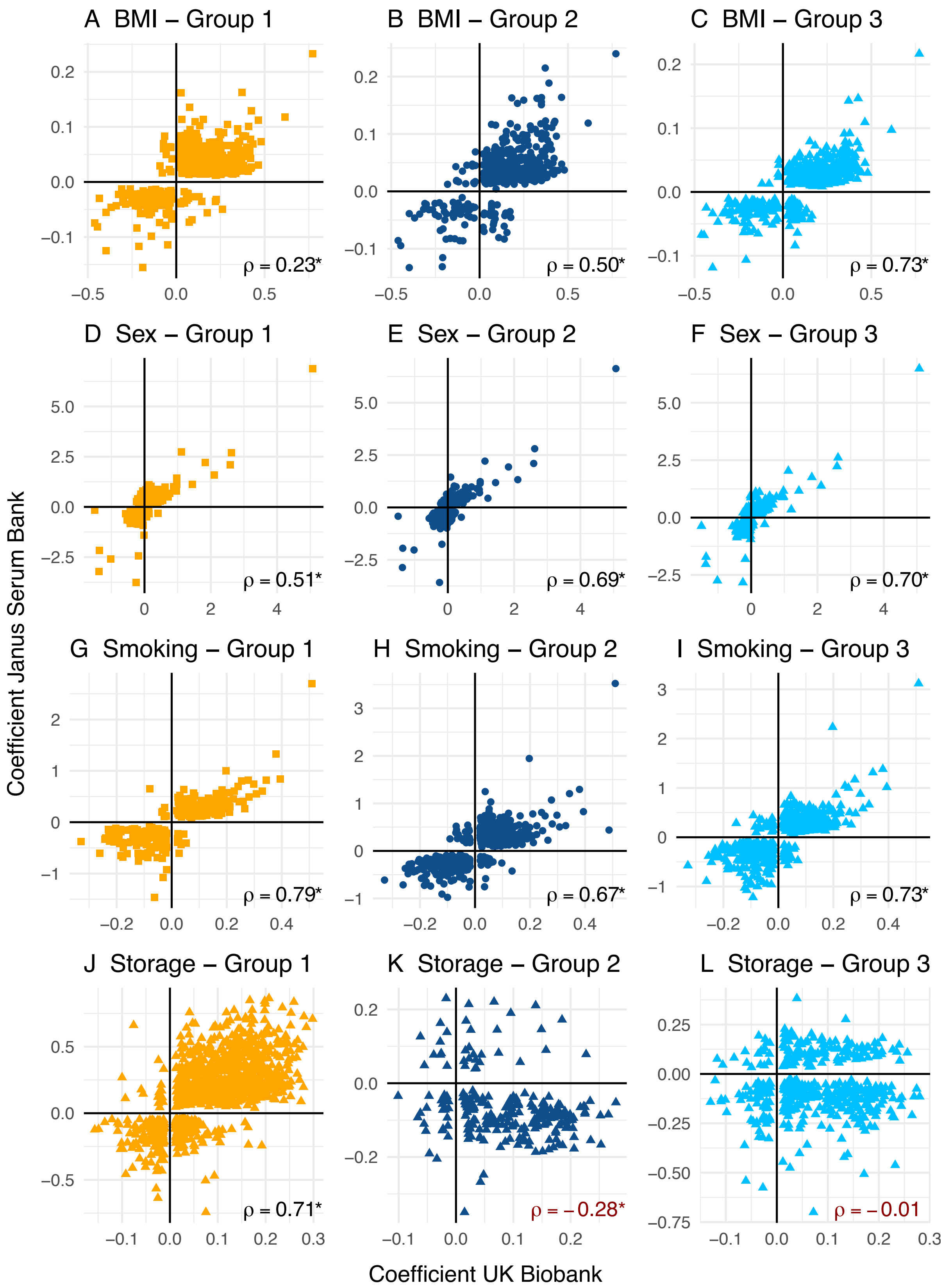

Supplementary Figure 3. Per collection group coefficients of association (Spearman rho) for those associations that are nominally significant (p-value < 0.05). UK Biobank reference coefficients are provided on the x-axis and the study samples coefficients for Group 1-3 on the respective y-axes A) Group 1 coefficients of association for BMI. B) Group 2 coefficients of association for BMI. C) Group 3 coefficients of association for BMI. D) Group 1 coefficients of association for sex. E) Group 2 coefficients of association for sex. F) Group 3 coefficients of association for sex. G) Group 1 coefficients of association for smoking status. H) Group 2 coefficients of association for smoking status. I) Group 3 coefficients of association for smoking status. J) Group 1 coefficients of association for storage time. K) Group 2 coefficients of association for storage time. L) Group 3 coefficients of association for storage time. Statistics for these data are provided in Supplementary Table 2.

Supplementary Table 1. List of statistics for individual assay targets for all models analysed. The list is provided as a separate file due to large table dimensions.

Supplementary Table 2. Comparison of the fractions of storage time affected proteins for the 20 % lowest expressed proteins and the 20 % highest expressed proteins.

	Associated to storage time	Not associated to storage time	Total
**Quintile 1 (lowest expressed)**	329 (56 %)	259 (44 %)	588
**Quintile 2 (highest expressed)**	327 (56 %)	260 (44 %)	587

Supplementary Table 3. Spearman correlation coefficients (rho) for the effect sizes for BMI, sex, smoking status and storage time for the collection groups in the current study and the reference data based on studies performed on samples of the UK Biobank (3, 4). Correlations were calculated for the coefficients of those assay targets that were nominally significant in the respective stratified collection group analyses and the UK Biobank. The visual representation of the effect sizes of the phenotypes investigated in the strata of collection groups and the UK Biobank are provided in Supplementary Figure 3.

Phenotype	Group 1 versus UKB	Group 2 versus UKB	Group 3 versus UKB
	rho (p-value, N proteins)	rho (p-value, N proteins)	rho (p-value, N proteins)
**BMI**	0.23 (< 0.001, 731)	0.50 (< 0.001, 524)	0.73 (< 0.001, 472)
**Sex**	0.51 (< 0.001, 821)	0.69 (< 0.001, 532)	0.70 (< 0.001, 565)
**Smoking status**	0.79 (< 0.001, 336)	0.67 (< 0.001, 596)	0.73 (< 0.001, 552)
**Storage**	0.71 (< 0.001, 1279)	-0.28 (< 0.001, 239)	-0.01 (0.86, 472)

UKB – UK Biobank
